# Supplementary material for: Antimicrobial prescription practices for outpatients with acute respiratory tract infections: A retrospective, multicenter, medical record-based study
Source: PLoS One. 2021 Nov 12;16(11):e0259633. doi: 10.1371/journal.pone.0259633 (PMC8589193; doi:10.1371/journal.pone.0259633)
Supplement: Attachment — Submitted filename: Response to Reviewers.docx [file pone.0259633.s002.docx]

29th/September/2021

*Dear Prof. Simon Clegg, PhD*

*Academic Editor*

*PLOS ONE*

Ref: PONE-D-21-23291-R1

Antimicrobial Prescription Practice for Outpatients with Acute Respiratory Tract Infections: A Retrospective, Multicenter, Medical Record-based Study

We hereby resubmit our above-named manuscript for reconsideration for publication in *PLOS ONE*. We have carefully considered all of the enclosed comments and addressed them as thoroughly as possible. Point-by-point responses to the reviewers’ comments are given below. The corrected sentences are noted with track changes in the revised version.

We hope you will now find our revised manuscript finally acceptable for publication in *PLOS ONE*.

Sincerely yours,

Hideharu Hagiya, M.D., Ph.D.

Department of General Medicine, Okayama University Graduate School of Medicine, Dentistry and Pharmaceutical Sciences, 2-5-1 Shikata-cho, Kita-ku, Okayama 700-8558, Japan

Tel: +81-86-235-7342	Fax: +81-86-235-7345

E-mail: [hagiya@okayama-u.ac.jp](about:blank)

**Comment from Reviewer #1**

This study presents antibiotic prescription practices for acute respiratory tract infections across 7 medical institutions in Japan. Whilst this study is certainly timely and highly important, if affords further elaboration and statistical analyses, and more in-depth argumentation to better understand prescription practices across the 7 institutions and for various factors of interest.

**Response**

We greatly appreciate your effort to review our study. We have provided point-by-point comments below.

**Title:**
• Comprehensive title, however, would remove the superscript (number 1) as it is just confusing.

**Response**

As per your indication, we have deleted the superscript.

• Would also consider slight modification. This study is evaluation antibiotic prescription practices specifically therefore I would be more specific in the title and avoid the term antimicrobial which encompasses all antimicrobial drugs, not solely antibiotics. Would therefore suggest slight rephrasing to: “Antibiotic Prescription Practices for Outpatients with Acute Respiratory Tract Infections: A Retrospective, Multicenter, Medical Record-based Study”

**Response**

Thank you for your advice. In general, it is usual to use the term “antimicrobials”, rather than “antibiotics”, in this specialized area of infectious disease. Also, it is obvious that we are dealing with “antibiotics” in this study when readers go through the manuscript. Thus, we would like to remain “antimicrobials” as it is.

**Background:**
Overall background is structured well but can be made more concise and language should be revised. Please see my specific comments below:
• “In the guidelines, the use of antimicrobials for the treatment of ARTIs is not recommended [7,8].” This claim is untrue. Prescription of antibiotics for ARTIs is often unnecessary and not in accordance to guidelines, however there are circumstances, even in the guidelines where antibiotics are recommended, if not for immediate use, at least for delayed use.

**Response**

Thank you for your advice. According to your comment, we have changed the sentence as follows; *According to the guidelines, prescription of antimicrobials is often unnecessary for ARTIs treatment, although there are actually cases that require a delayed prescription [7,8]* (Line 67-68)

• “However, in reality, antimicrobials are often prescribed in the clinical setting in Japan partially because of requests from patients or due to physicians’ anxiety.” What evidence do you have to support this claim? Is this anecdotal evidence or are there reports/studies that have found patient demand and physician anxiety to impact antibiotic prescription? Is it really a result of physician anxiety? Several other factors have been identified in the literature, including uncertainty, lack of access to diagnostic testing, perceived pressure from patients, knowledge and awareness, etc.

**Response**

This sentence is just an anecdotal comment from us, without a scientific analysis. Thus, we have changed this sentence as follows; *However, in reality, we, as Japanese clinicians, frequently witness antimicrobials being prescribed in outpatient settings, partially because of requests from patients or due to physicians’ anxiety.* (Line 68-70)

• “Approximately, in half of these visits antimicrobials were prescribed (532.4 per 1000 person-years; 99% CI, 531.6–533.3)”: provide the exact number.

**Response**

These numbers are the exactly given in the literature. Please go through it. We have modified the sentence so as it is more understandable; *antimicrobials were prescribed in half of these visits (532.4 per 1000 person-years; 99% CI, 531.6–533.3) [11]* (Line 77-78)

• “Most of the antimicrobials prescribed for ARTIs were broad-spectrum oral formulations including cephalosporins (41.9%; third-generation cephalosporins, 97.3%), macrolides (32.8%), and fluoroquinolones (14.7%) [12].” The way data for cephalosporins are presented is very confusing. Please reframe.

**Response**

We rephrased the corresponding sentence as follows; *Most of the antimicrobials prescribed for ARTIs were broad-spectrum oral formulations including cephalosporins (41.9%, of which third-generation cephalosporins accounted for 97.3%), macrolides (32.8%), and fluoroquinolones (14.7%) [12]* (Line 78-80).

• “Another Japanese study revealed that despite the decreasing trend, the issue of inappropriate antibiotic prescriptions for non-bacterial ARTIs persisted during the study period from 2012 to 2017 [13].” What were the prescription rates in this study and how do they compare to others? Did they assess guideline-concordant antibiotic prescription?

• “The proportion of antimicrobial prescriptions for ARTIs in Japan is reportedly higher than that in other countries [15–17].” Which countries? What are the rates there and how do they compare to Japanese data?

**Response**

The monthly antibiotics prescription rate was 31.65 per 100 consultations for nonbacterial ARTIs. They have just retrospectively collected data on the antibiotics prescribing and thus the study was not an evaluation for the Guideline compliance rate.

The prescribing rate cannot be simply compared to other preceding studies since the claims data is not identical each other. However, we introduced the results of previous study from the United States as examples.

We have changed the sentences as follows; *Despite the higher antimicrobial prescription rates in Japan, downward trends have been reported. A retrospective, observational study using longitudinal, administrative claims data revealed that a mean monthly antimicrobial prescription rate for nonbacterial-ARTIs was 31.65 per 100 visits between April 2012 and June 2017 [14]. The antimicrobial prescription rate decreased by 19.2% during the study period; however, there was no remarkable trend change compared to other countries. For instance, previous national data in the United States (1995 to 2006) suggested that ARTIs-associated antimicrobial prescriptions decreased by 36% among children younger than 5 years and by 18% among persons aged 5 years or older [15]. According to another analysis of nationally representative data in the United States (2000 to 2010), antimicrobial prescription for ARTIs decreased by 57% among children and adolescents (<18 years) and 38% among adults (18 to 64 years), although there was no certain trend among those aged ≥65 years [16]. Thus, it is possible that the decrease in the proportion of antimicrobial prescriptions for ARTIs in Japan may be further accelerated.* (Line 84-95)

• Please better explain why insurance claims data is less credible than your methodology. Rather than just stating that it is less credible, explain why. Is it because it is biased towards more several ill patients? Do all patients file health insurance claims in Japan?

**Response**

The health insurance claims data lacks credibility because it is accumulated without clinical records. We have added this sentence in Line 97-99.

All the clinical data on Japanese patients is not filed at all.

**Methods:**
• Are there any differences in the patient populations across the 7 different institutions? For example, do some cater for urban whereas others cater for more rural populations? I would appreciate more information about the clinics. Are the capacities different? A busier clinic might have higher inappropriate prescribing rates just because doctors do not have the time to educate patients and think through their ordering practices as smaller clinics might.

**Response**

All these institutes are located in the rural areas, and the patient populations are almost identical. The first four institutes (Marugame, Kasaoka, Tamano, and Kaneda) are regional general hospitals with inpatient beds, while the Niimi National Health Insurance Clinics are no-bedded outpatient clinics. (Line 108-111)

• I would also like to know more about the prescription laws in Japan and who is authorized to prescribe. Are the prescriptions you are evaluating only provided by medical doctors? Or can nurse practitioners also prescribe and if so why not evaluate whether there are any statistically significant differences in the antibiotic prescribing patterns of the two, i.e. nurse practitioners versus medical doctors. You can also be more specific regarding what kind of medical doctors’ prescriptions you are assessing. Are they general practitioners? Are they specialists? Are they respiratory specialists?

**Response**

In Japan, only medical doctors are authorized to prescribe antimicrobials, but not nurse practitioners. The antimicrobial prescriptions included in this study were not limited to either general practitioners or any organ specialists. (Line 128-129)

• I think you need to explicitly motivate why you excluded all patients under 18 years of age.

**Response**

In Japan, it is usual to deem individuals aged >18 years as adults. Thus we excluded those whose ages were 18 years or less.

• Why did you restrict your analysis to this small list of antibacterials? Are J01C antibacterials never prescribed in Japan for example? If so they should have definitely been captured as a broad-spectrum prescription. Why have they been excluded entirely? What about J01D antibacterials? You restricted your data capture to 3rd-gen cephalosporins and faropenem specifically, but what about other ‘other beta-lactam penicillins’ such as 1st and 2nd-gen cephalosporins?

**Response**

I think the reviewer misunderstand ICD-10 codes and ATC classification. In the 2^nd^ paragraph of the Method section, we refer to the classification of ICD-10 codes to define ARTIs by referring to the previous, related studies [references 6,11,17]. For the classification of antibiotics, we referred to other reports [references 15, 18]. (Line 133-135)

• You collected data on age, sex, presence of upper or lower (cough and sputum expectoration) respiratory symptoms, clinical diagnosis defined by the ICD-10 codes, and antibiotic prescription. You also further categorized age groups. Why collect and categorize these data but then not use them for more in-depth analysis?

**Response**

We have utilized the collected data of age to stratified the age groups. Presence of upper or lower (cough and sputum expectoration) respiratory symptoms were used to confirm whether the diagnosis given by the ICD-10 codes was clinically endorsed or not. We did not stratify the data by sex; however, otherwise, we have used the collected data appropriately.

• You excluded patients with IV antibiotic therapy. Does that mean that you only included patients who received oral antibiotics? In that case, please specify that. Right now it seems like you included all patients with any antibiotic prescription for specific ICD-10 codes, except for IV antibiotic prescriptions.

**Response**

We excluded those who received intravenous antimicrobial therapy to include patients prescribed with oral antimicrobials alone. (Line 158-159)

*> Right now it seems like you included all patients with any antibiotic prescription for specific ICD-10 codes, except for IV antibiotic prescriptions.*

Yes, that is true for our study.

• Statistical analysis is poorly written. Lacks detail. Even if analysis was descriptive, I would like to know which specific methods you used.

**Response**

We have totally amended the statistical method parts. Please go through the revised paragraph (Line 137-158)

• Regarding your outcomes, this is the first time that we learn that you are also including other drugs prescribed for ARTIs, i.e. not just antibiotics. You should also motivate why your primary outcome is the common cold specifically.

**Response**

Common cold is the representative of ARTIs, which is known to be caused by viral etiologies in most of the cases. Actually our main concern is the antibiotics prescription rates for the common cold. We are sorry for the insufficient explanation. We have amended the final sentence of the Introduction part as follows; *The present study aimed to determine the proportion of antimicrobial prescriptions for ARTIs, especially for the common cold, by directly examining the medical records.* (Line 100-101)

• Were the prescriptions you analyzed all for immediate use? Can you differentiate whether they were for delayed use or not?

**Response**

This is important issue, however, unfortunately, it is impossible to determine the prescribed antibiotics were for immediate use or delayed use. We have added this sentence at Line 310-311.

**Results:**
• Please specific the number of cases for all clinics (split the Niimi National Health Insurance Clinics into the 3 different clinics). This comment pertains figure 1 (which I think can be deleted), table 1 and the text. If you would like to keep that group of clinics together, then at least you need to explain why because in the methods you say that you analyze the prescription practices of 7 clinics and then you seemingly restrict to 5 which makes it confusing.

**Response**

Thank you for your advice. As the total number of patients in the Niimi National Health Insurance Clinics is only 51. Thus there is no merit to spilt data more in detail. We therefore explained that we have collected data from “five” clinics, not seven clinics, through the manuscript.

• First paragraph of the results can actually move to the methods section in my opinion, when you describe inclusion and exclusion criteria. Please also refrain from inserting number in the text. You should place them in brackets with “n=” before each number, e.g. “data of 3,940 patients were analyzed: Marugame Medical Center (n=996); Kasaoka City Hospital (n=1433)”, etc.

**Response**

It is natural for us to place this first paragraph as it is. We expect your understanding. While, the way describing the number of cases were amended as recommended.

• Table 1: Please change title of the table (no informative). Also, lots of details are lacking. Make sure to provide them. For example, insert all percentages. Do not just present the ICD codes without explaining what they are; not all readers are familiar with them but will certainly be familiar with the description.

**Response**

The title was changed to “*Numbers and percentages of background data and diagnosis of the cases in each medical institute by age groups.*”. Also, we have provided percentages in each figure.

The ICD-10 codes are explained as follows in the Table foot note; *The International Classification of Diseases (ICD-10) codes were endorsed in May 1990 by the Forty-third World Health Assembly to develop the diagnostic classification standard for all clinical and research purposes.*

• No need to label categorized age with “adult, early elderly, late elderly”. Seems purely subjective labelling and unnecessary. Also please make sure not to refer to these labels in the text but rather you the age ranges; again for the sake of clarity. Please will not remember the age ranges for each of the labels you provided.

**Response**

We unlabeled the age groups. As per the advice, we deleted the labelling from the text entirely.

• You present your results using mean and SD. Are data normally distributed? Is this the correct summary statistic or should you have presented medians and IQRs? This is unclear since your description of your statistical analysis in the methods section is vague.

**Response**

We appreciate your comment. We cannot demonstrate all the data is normally distributed in this study. Thus, we summarized the data with using median and IQRs, instead of mean and SD. Additionally, we have newly made it clear that continuous variables were summarized with median and IQRs in the Method section. (Line 142-143)

• Why collect data on symptoms but not present it here?

**Response**

We applied the symptoms data in Table 2 to confirm that patients diagnosed with common cold had two or more or organ-related symptoms.

• In think you afford running statistical models on the data and not just keep it at a descriptive level. Are there statically significant prescription practices across the clinics, across age groups, across the various ICD-10 diagnosis? Are there statistically significant differences in prescribing practices if more than 1 clinical manifestation was involved?

**Response**

We appreciate your recommendation. In this study, we are not interested in the comparison of antimicrobial prescription among the five medical institutes and among the cases with different ICD-10 diagnosis. Our primary aim of the present study is to investigate the proportion of antimicrobial prescriptions for ARTIs, especially the common cold. We thus additionally performed a statistical analysis for such purpose. Strength of this study is to collect clinical data endorsing the diagnosis of common cold, therefore, we stratified the eligible cases by the numbers of clinical manifestations suggesting common cold; “Three respiratory regions”, “Two respiratory regions”, and “Two or more respiratory regions”, as have done in the first-submitted manuscript. The results are given in the newly-formatted Table 2 and the second paragraph of the Discussion.

• I find the way you have divided the total number of clinical manifestations to be confusing. You write all involved, 2 involved and >= 2 involved. Where there never just 1 clinical manifestation noted? And what’s the difference between all involved and >= 2 involved?

**Response**

Sorry for the confusing description. As written above, we have renamed the term as either of “Three respiratory regions”, “Two respiratory regions”, and “Two or more respiratory regions”. Also, the explanations are amended as in the Table footnote as follows;

**"Three respiratory regions” denotes that clinical manifestations of all three distinct respiratory tract regions (nasal, pharyngo-laryngeal, and bronchial) were described in the medical records. **"Two respiratory regions” and ***"Two or more respiratory regions” denote that as it appears, clinical manifestations of each of*

*the two and two or more of the respiratory tract regions were described.*

Because we would like to confirm there were clinical manifestations relating to ARTIs in the eligible patients in this study, we were interested in the numbers of organs (either each of nasal, pharyngo-laryngeal, or bronchial areas). Patients with “Three respiratory regions” were those who manifested all the three distinct respiratory tract regions. While, patients with " Two or more respiratory regions” we those who manifested two or three distinct respiratory tract regions.

• In table 2, what does “others” refer to? Other antibacterials or other drugs such as symptomatic relief medications?

**Response**

“others” referred to the other antimicrobials. We have amended it as appropriate.

• In tables 2 and 3 why split the data by age if you do not statistically measure differences between the various age groups? Also, you now include 16 and 17 year old patients. Were they or were they not included? You also need to describe what kind of data you are presenting in these tables. In the legend you say that CI refers to confidence interval but it is not immediately obvious in the table which results you are referring to. Although it can be assumed, you need to specify.

**Response**

As written above, we performed the statistical analysis to compare the antimicrobial prescription among the age group (see the new Table 2).

We included those aged 18 years and more in this study, not 16 and 17 year-old patients. The age category was appropriately revised as “18-64 years”.

We have improved the Table contents considering the readability.

**Discussion:**
• Whilst some good points are raised the discussion lacks depth and sometimes lacks flow. Please try re-writing it to bring arguments together in a more comprehensive manner.

**Response**

Thank you for your comment on our Discussion. We have amended it with caution so as it conveys meaningful points to readers, with better flow.

• First paragraph refers to studies and previous literature but lacks references. And how do your data compare? How much lower that what other studies have found?

**Response**

Our data showed antimicrobial prescribing rates for common cold were less than 20% in all the age group, which was much lower than previous studies based on health insurance claims data (Reference 9, 11, 13). We have provided the reference numbers as appropriate. Please see the second paragraph of the Discussion where the details are given.

• Please consider sentence structure in the 1st paragraph. I would also be hesitant to claim that “broad spectrum agents were the most frequently prescribed antimicrobials” because from your methods your data capture seemed to be restricted to specific antibiotics/antibiotic classes and so any other narrow-spectrum antibiotics may have not been captured, as well as any other broad-spectrum antibacterials that for some reason were excluded from this study (not immediately clear as to why this decision was made).

**Response**

As already described in the Data collection paragraph of the Method section, we considered third-generation cephalosporins, macrolides, fluoroquinolones, and faropenem as broad-spectrum antimicrobials, while deemed penicillins as narrow-spectrum antimicrobials, according to previous studies [reference 15,18]. (Line 133-135) As clearly shown in the Table 2, if prescribed, the antibiotics were those included in the broad-spectrum drugs. We did not restrict data into these antibiotics alone, and opened all the data collected from the survey. Truly, the broad-spectrum antibiotics, such as third-generation cephalosporins, macrolides, fluoroquinolones, and faropenem were prescribed with high proportions. See the newly-given Table 2.

• In the Kimura study, the trend decreased by 19.2%, but from what percentage? And what was the final percentage in 2017?

**Response**

According to the study, the antibiotics prescriptions for nonbacterial ARTIs in 2012 and 2017 were 34.4 and 27.8, respectively. (Line 252-254)

• There are a few studies from southern Europe that describe high broad-spectrum antibiotic use and uncertainty avoidance that I think can be referred to in the discussion.

**Response**

We have already cited many related articles and the total number of the citation is up to 30. We thus consider it is enough to cite previous studies at the present manuscript.

• Delayed antibiotic prescription strategies are mentioned in one sentence. I would like you to expand upon this and explain why this strategy could be beneficial in your setting. Is this something that is commonly practiced or not? Is it worth investigating further?

**Response**

Thank you for your comment and advice. We have expanded the discussion as in Line 287-2298 by incorporating the data on the number needed to treat for ARTIs by antibiotics.

• Something else you could consider noting is that doctors may have adjusted their diagnosis to best fit their ordering behavior. The advantage of your study design is that data were pulled retrospectively and that doctors were not aware that their prescription practices were to be analyzed.

**Response**

Thank you for reminding us of this point. We have added this as the 4^th^ limitation of this study; *Fourth, the ICD-10 codes given in the medical records might be labelled for convenience and in a manner that was not based on an actual diagnosis, so as to best fit their antimicrobial prescriptions. This could have been true in some cases, but cannot be reviewed at this point.* (Line 311-314)

• Finally, I think the fact that a good percentage of the patients received antibiotics for the common cold should not be overlooked. The conclusion to me is more positive than it should be. Whilst the prescription rates may be lower than other settings, they are not low enough, and certainly not for the common cold where prescription should be down at 0%. It is good that you highlight however that broad-spectrum antibiotic use is high and needs to be addressed.

**Response**

Thank you for understanding our conclusion. As mentioned, the antimicrobial prescription for common cold should be much more reduced. We feel that now is on the way to a positive future with such good practice. We have amended the conclusion paragraph according to your advice as follows;

*In conclusion, our findings from this clinical data-based study suggest a favorable reduction in the amount of antimicrobials prescribed for outpatients presenting with a common cold. However, the antimicrobial prescription for the common cold should be further reduced because it is caused by viral etiologies and resolves without specific treatment, usually in a few days. In addition, broad-spectrum antimicrobials are still prescribed for the common cold at high rates, spurring the need for future studies focusing on the choice of drugs. These findings may be useful for health policy makers as a benchmark for monitoring the effectiveness of AMS promotion strategies in Japan.*

**General comments:**
• Language: Please take the time to do an extensive review of the manuscript’s language. Whilst the manuscript is well-written overall, it can be made more concise and there are some grammatical and sentence-structure issues that need correcting before publication. Arguments in the discussion can also flow better. I would also avoid sweeping statements and using words such as “menace”.

**Response**

Before resubmission, we have again made the manuscript being checked by an English-native. Please again go through the text whether it has reached an acceptable level in terms of English grammar.

**Comment from Reviewer #2**

**Comment #1**

The data from this study suggest that a lower percentage of ARTI cases (common cold) are given antibiotics (12%) than in 2018, but that ~90% of these are broad spectrum. This corresponds with your conclusions, which report this downwards trend and also the discussion of how to reduce broad spectrum usage

**Response #1**

Thank you for your comment. We appreciate your understanding for our study conclusion. Beyond our expectation, the antimicrobial prescribing rates for outpatients with ARTIs were limited to 12.7%. However, as in the conclusion, the broad-spectrum antimicrobials are still frequently prescribed, which urges us to promote further antimicrobial stewardship in the outpatient setting.

**Comment #2**

The only analysis you use is confidence intervals, more could have been made of the data as there was no use of tests for identifying differences between groups, e.g. age groups, types of drugs used and for what infection type, which could have identified some more, perhaps interesting, results. If you had data from previous surveys, this could have also been compared.

**Response #2**

Thank you for your advice. As per the recommendation by you and another reviewer, we additionally performed the statistical analysis to compare the antimicrobial prescription among the age groups. The results are given in the new Table 2. This time, we are not intended to antimicrobial prescribing among each medical institute.

**Comment #3**

According to your declaration, you have made all data available

**Response #3**

Our data is available if requesting to a corresponding author.

**Comment #4**

You have written this in clear and understandable language, although whilst the discussion is clear that ARTIs are mainly viral, this could be made clearer in the introduction

**Response #4**

We have already provided the fact that the majority of the ARTIs is virally caused (Line 65-66).

*Thank you for your review.*
