# Supplementary material for: Antimicrobial prescription practices for outpatients with acute respiratory tract infections: A retrospective, multicenter, medical record-based study
Source: PLoS One. 2021 Nov 12;16(11):e0259633. doi: 10.1371/journal.pone.0259633 (PMC8589193; doi:10.1371/journal.pone.0259633)
Supplement: Attachment — Submitted filename: Response to Reviewers.docx [file pone.0259633.s003.docx]

20th/October/2021

*Dear Prof. Simon Clegg, PhD*

*Academic Editor*

*PLOS ONE*

Ref: PONE-D-21-23291-**R2**

Antimicrobial Prescription Practice for Outpatients with Acute Respiratory Tract Infections: A Retrospective, Multicenter, Medical Record-based Study

We hereby resubmit our above-named manuscript for reconsideration for publication in *PLOS ONE*. We have carefully considered all of the enclosed comments and addressed them as thoroughly as possible. Point-by-point responses to the reviewers’ comments are given below. The corrected sentences are noted with track changes in the revised version.

We hope you will now find our revised manuscript finally acceptable for publication in *PLOS ONE*.

Sincerely yours,

Hideharu Hagiya, M.D., Ph.D.

Department of General Medicine, Okayama University Graduate School of Medicine, Dentistry and Pharmaceutical Sciences, 2-5-1 Shikata-cho, Kita-ku, Okayama 700-8558, Japan

Tel: +81-86-235-7342	Fax: +81-86-235-7345

E-mail: [hagiya@okayama-u.ac.jp](about:blank)

**Comment from Reviewer #1**

**Reviewer #1**

Thank you for addressing my comments thoroughly and appropriately, and for giving me the opportunity to review this paper. I have just two remaining minor comments regarding the tables.

For table 1, as per my previous comment, please include the full names of the ICD-10 codes in the footnote, i.e. acute nasopharyngitis (J00), acute sinusitis (J01), acute pharyngitis (J02), acute tonsillitis (J03), acute laryngitis and tracheitis (J04), etc.... For someone not too familiar with the codes, it is important that that information is directly available in the table.

**Response**

We totally agree with your advice and provided the required information in the footnote of the Table 1.

In table 3, the categorization of clinical manifestations is still ambiguous to me. Whats the difference between two or more respiratory regions and three respiratory regions? Were there no patients in any of the age groups that presented with only 1 respiratory region affected? What is the miscellaneous column representing? I don't see any data presented for it in this table (neither in table 4). Why not remove that column from both tables?

**Response**

Thank you for your comment again. Usually, acute respiratory tract infections (ARTIs) causes a variety of manifestations, since pathogens infects with a broad part of the respiratory tracts (nasal, throat, and bronchi). Therefore, we are interested whether the respiratory tracts of the patients were widely involved or not. “Three respiratory regions” means all these respiratory tracts are involved. “Two respiratory regions” denotes two of the three respiratory tracts are involved. “Two or more respiratory regions” is a total of these cases. The last pattern may not be necessarily needed; however, we are intended to make it clear all these patient patterns.

Patients with only 1 respiratory region were excluded from this Table, because they were possibly infected with bacterial pathogens that require antimicrobial treatment, to which we are not interested in this study. Similarly, data for miscellaneous cases is not main purpose of this study, and thus, we dared not present such cases in the table. Hoping your understanding.

*Thank you for your review.*

**Reviewer #3**

This is a very nicely written and interesting manuscript, for which I only have very minor comments

**Response**

We greatly appreciate your comment. Amendments were given appropriately as per your advice.

Line 40- 50.1% of cases (reword)]

**Response**

This was revised appropriately.

Line 68-69- In approximately half of these visits …. (reword)

**Response**

This was revised appropriately.

Line 91- I think doing a separate study on children would be of interest here too

**Response**

Thank you for your comment. We agree that another study specifically targeting children is warranted in a future.

Line 139-140- this reads slightly awkwardly and may read better as- There were no notable differences in the proportions of antimicrobial prescriptions in cases involving infection in all, two or one region of the respiratory tract (16.7%, 18.2% and 17.7% respectively).

**Response**

The corresponding sentence is rewritten. Please go through the Line 194-199.

Line 175- the higher the frequency of … (reword)

**Response**

This was revised appropriately.

Line 179- how much of this is an expectation due to age? So when a younger 70 year old went to the doctors, they would always get antibiotics, but the shift in thinking may affect this?

**Response**

It is very interesting point to be addressed. Patient’s age potentially affects their behaviors for seeking antibiotics. However, it cannot be estimated in our data. Instead, we referred that various background factors may influence on the antimicrobial prescribing as follows; *A potential influence of these factors may differ among medical situations, routine practices of individual clinicians, experiences and ages of patients, and societies with different cultural and healthcare backgrounds.*

Line 222- perhaps a comparison of this data to administration data maybe useful?

**Response**

We appreciate your recommendation. It might provide useful information, but this time, it is beyond our purpose. In future study, we will challenge for that.

Table 1- I think having the full data in for the J code maybe useful here

**Response**

We believe the present data of ICD-10 codes given in the Table 1 is enough to identify the respiratory diseases. Other similar articles also described their ICD-10 codes as we do.

But overall, a very good manuscript and a pleasure to read.

**Response**

Thank you for your review in this difficult time.
